# Generative artificial intelligence in healthcare from the perspective of digital media: Applications, opportunities and challenges

**DOI:** 10.1016/j.heliyon.2024.e32364

**Published:** 2024-06-05

**Authors:** Rui Xu, Zhong Wang

**Affiliations:** a School of Economics, Guangdong University of Technology, Guangzhou, China; b Key Laboratory of Digital Economy and Data Governance, Guangdong University of Technology, Guangzhou, China

**Keywords:** ChatGPT, Healthcare, Digital media, Applications, Opportunities, Challenges, Digital health, Generative artificial intelligence, Large language models, Artificial intelligence generated content

## Abstract

**Introduction:**

The emergence and application of generative artificial intelligence/large language models (hereafter GenAI LLMs) have the potential for significant impact on the healthcare industry. However, there is currently a lack of systematic research on GenAI LLMs in healthcare based on reliable data. This article aims to conduct an exploratory study of the application of GenAI LLMs (i.e., ChatGPT) in healthcare from the perspective of digital media (i.e., online news), including the application scenarios, potential opportunities, and challenges.

**Methods:**

This research used thematic qualitative text analysis in five steps: firstly, developing main topical categories based on relevant articles; secondly, encoding the search keywords using these categories; thirdly, conducting searches for news articles via Google ; fourthly, encoding the sub-categories using the elaborate category system; and finally, conducting category-based analysis and presenting the results. Natural language processing techniques, including the TermRaider and AntConc tool, were applied in the aforementioned steps to assist in text qualitative analysis. Additionally, this study built a framework, using for analyzing the above three topics, from the perspective of five different stakeholders, including healthcare demanders and providers.

**Results:**

This study summarizes 26 applications (e.g., provide medical advice, provide diagnosis and triage recommendations, provide mental health support, etc.), 21 opportunities (e.g., make healthcare more accessible, reduce healthcare costs, improve patients care, etc.), and 17 challenges (e.g., generate inaccurate/misleading/wrong answers, raise privacy concerns, lack of transparency, etc.), and analyzes the reasons for the formation of these key items and the links between the three research topics.

**Conclusions:**

The application of GenAI LLMs in healthcare is primarily focused on transforming the way healthcare demanders access medical services (i.e., making it more intelligent, refined, and humane) and optimizing the processes through which healthcare providers offer medical services (i.e., simplifying, ensuring timeliness, and reducing errors). As the application becomes more widespread and deepens, GenAI LLMs is expected to have a revolutionary impact on traditional healthcare service models, but it also inevitably raises ethical and security concerns. Furthermore, GenAI LLMs applied in healthcare is still in the initial stage, which can be accelerated from a specific healthcare field (e.g., mental health) or a specific mechanism (e.g., GenAI LLMs’ economic benefits allocation mechanism applied to healthcare) with empirical or clinical research.

## Highlights

• First systematic research of ChatGPT in healthcare with reliable data sources.
• Text analysis of articles derived from influential digital media.
• Summarization of 26 applications, 21 opportunities, and 17 challenges of ChatGPT in digital health age.

## 1 Introduction

ChatGPT has led the revolutionary development of generative artificial intelligence/large language models (hereafter, GenAI LLMs) [[1], [2], [3]] and has gradually been applied to important fields such as healthcare [4,5]. On November 30th, 2022, Open AI officially released ChatGPT1.0, which quickly became the most rapidly growing, widely used, and industry-spanning digital product in history [6], demonstrating the popularity and powerful influence of ChatGPT. ChatGPT is a GenAI LLMs that autonomously generates response text through machine learning training [2,7], representing the new stage in the development of GenAI LLMs [2], which is markedly different from analytical AI (e.g., forecasting the Estimated Time of Arrival of your delivery or predicting which TikTok video to show you next) that existed before. The greatest characteristic of GenAI LLMs is its ability to complete creative work, and even excel in some aspects over humans [3,8], which many academics and experts consider a revolutionary development in AI [9]. The development of a new product or field depends on the promotion of practical applications, and GenAI LLMs is no exception. In the nearly five months since ChatGPT was released, it has been applied or is being researched for application in education [10], urban management [11], economic research [12], climate governance [13], academic publication [14], and other fields, demonstrating huge potential for development and vast prospects for application. Among them, the healthcare field has become an important field for ChatGPT application due to the inevitable digital transformation and intelligent transformation of healthcare [15], as well as huge scale and broad market of healthcare [16]; the healthcare field also presents an important research area for how ChatGPT should be applied due to its involvement with patients as a special demographic and patient data as a highly-sensitive data [17].

In the current digital age, the development of GenAI LLMs represented by ChatGPT is dynamically evolving [18]. The application of ChatGPT, especially in the healthcare field, is mostly in the exploratory stage [4,19], and relatively few reference cases are available. However, reference cases are indispensable for academic papers to have sufficient validity and reliability, which leads to the limitations of comment articles. We must make trade-offs among the timeliness, reliability, and availability of data. Fortunately, the research perspective of digital media can meet these requirements for the following reasons. On the one hand, digital media, especially online news, has the characteristics of closely following current affairs and reporting cutting-edge dynamics [20], which can well meet the requirements of data timeliness. On the other hand, by appropriately screening web news with suitable criteria, researchers can obtain more news cases, more comprehensive news viewpoints, and more in-depth news reporting [21]. There have been precedents for exploring new fields in healthcare from the perspective of digital media, such as studying public participation in health science discussions during the COVID-19 pandemic [22], defining the connotation of digital health [23], and studying young adults’ sexual health [24].

This research, thus from the perspective of digital media, aims to clarify application scenarios of GenAI LLMs in healthcare, followed by analyzing and discussing potential opportunities and risks based on different scenarios, subsequently forming a systematic and framework-oriented study. In view of research on GenAI LLMs’ application to healthcare is currently mainly in the exploratory stage, clarifying these three topics of GenAI LLMs in healthcare has framework-guiding significance and foundation-exploring value [25]. This article primarily emphasizes the application of GenAI LLMs in healthcare rather than the technology of GenAI LLMs itself.

This paper is organized as follows. Section 2 provides a literature review on the relevant research. Next the research method is introduced in detail, and the statistical features of the search results are explained. Section 4 conducts text analysis on the content of web news and presents the research results. Section 5 conducts in-depth discussion and analysis of the research results. The paper concludes with a summary and discussion of future research directions.

## 2 Literature review

### 2.1 ChatGPT and GenAI LLMs

ChatGPT (chat generative pre-trained transformer) is an AI chatbot, a GenAI LLM, and a machine-learning system designed to reach a level of autonomous dialogue and generate valuable language and replies through massive text training [26]. With its human-like AI conversation style, ChatGPT has rapidly become popular worldwide, and has been first applied in scientific research fields (for summarizing literature, drafting and improving papers, as well as identifying research gaps and writing computer code). On December 16, 2022, half a month after the release of ChatGPT, the first academic paper with ChatGPT as the author was published [27], which triggered extensive discussions about whether ChatGPT can be a reliable author [28] and sparked a new wave of ChatGPT applications. ChatGPT has been updated to version 4.0 as of the completion of this article, with performance continuously optimized and greatly improved [18,29].

ChatGPT represents a new stage of AI development-- GenAI LLMs [30]. GenAI LLMs can produce new and creative content including images, texts, music, video, and other forms of design [31]. These capabilities have been significantly enhanced and have gained practical value due to GenAI LLMs’ high-cost investment and large-scale training, making it possible for GenAI LLMs to replace some jobs that previously could only be done by humans (e.g., writing, reasoning, imaging) [6,8]. Therefore, GenAI LLMs will have a profound impact on the development of science and whole society, not just in the initial scientific research fields [7,32]. Undoubtedly, GenAI LLMs will bring benefits to people in some ways, but it will also bring complex and challenging issues such as ethics [33], privacy [34], especially in life-and-death areas such as healthcare. Therefore, it is highly practical and valuable to promote technological development, industry self-regulation, and legal supervision through relevant research [1]. However, there is limited systematic research on the application scenarios and potential impacts of ChatGPT, partly due to a lack of data support.

### 2.2 ChatGPT in healthcare

Existing research on ChatGPT in healthcare concentrates on the following four aspects. Firstly, initial research provides an introduction or summary of ChatGPT in healthcare, mainly in the form of literature reviews [[35], [36], [37]] (see Table 1) and comments/viewpoints [16,38,39]. Secondly, a larger proportion of studies explore the development of ChatGPT in a specific field of healthcare, such as medical writing [40], medical advice [41], medical education [42]. Thirdly, small proportion analysis has been done on the impact of ChatGPT in healthcare, such as the potential impact of ChatGPT in clinical and translational medicine [43], the impact in simplified radiology reports [44], the impact in health services as a virtual doctor [45]. Newly emerging studies begin to discuss the utility of ChatGPT [46,47], the accuracy and reliability of ChatGPT responses [48], the feasibility of ChatGPT [19], applicability of ChatGPT [49], the robustness of ChatGPT [50], when applied ChatGPT to healthcare.

**Table 1:** Profile of reviews of ChatGPT in healthcare.

Field	Types	Examples	Quantity
ChatGPT applications in healthcare	Systematic reviews	[36,46,51]	27
	Scoping reviews	[47,52]	23
	Narrative reviews	[37]	5
ChatGPT opportunities in healthcare	Systematic reviews	[53]	10
	Scoping reviews	[54,55]	5
	Narrative reviews	[56]	2
ChatGPT challenges in healthcare	Systematic reviews	[17,35,53]	9
	Scoping reviews	[54]	2
	Narrative reviews	[57]	1

Note: (1) The methodology employed to identify these reviews is elaborated in *3.1 Research Method & Initial work with the text* within this paper. (2) For a comprehensive understanding of the various review types and methods for differentiation, reference was made to PubMed (URL: https://pubmed.ncbi.nlm.nih.gov/36414363/, accessed on December 28, 2023). (3) The data presented in this table was retrieved on December 28, 2023, while developing the supplementary table for enhancement purposes.

The research on ChatGPT healthcare applications mainly focuses on two aspects. Firstly, most studies focus on applying ChatGPT to a specific scenario of healthcare, including writing patient clinic letters [58], seeking a vigilant and robust healthcare system [59], applying in translational medicine [60], exploring the future of nursing [61]. Secondly, few research has investigated overall scenarios in which ChatGPT is used [19].

The research on ChatGPT healthcare opportunities mainly focuses on a specific field, such as mental health [62], obstetrics and gynecology [56], and many other fields. For example, implementing ChatGPT in medical writing has the potential to standardize and improve the accessibility of medical information, enhancing patient participation and health outcomes [40,55]; equipped LLMs to perform complex clinical operations have the potential to revolutionize dental diagnosis and treatment [63]; applying ChatGPT in gastroenterology has significant potential in facilitating patient - physician interactions and managing diseases [52].

The research on ChatGPT healthcare challenges concentrates on ethical challenges [64], as well as challenges in a specific field, such as mental health [62], medical education [65], clinical settings and medical journalism [56]. To be detailed, GPT triggers privacy issues within medical ethics, data interpretation, accountability [66]; ChatGPT could cause potential legal ethics concerns arise from the unclear allocation of responsibility when patient harm occurs and from potential breaches of patient privacy due to data collection [57]; ChatGPT also raises algorithmic ethics concerns about algorithmic bias, responsibility, transparency and explainability, as well as validation and evaluation [67].

### 2.3 Summary

There are relatively few systematic studies of ChatGPT in healthcare with reliable data sources indicated in the literature review above. Therefore, a new perspective is urgently needed to fill the research gap mentioned above.

## 3 Analytical method and materials

### 3.1 Research method

This article adopted thematic qualitative text analysis to conduct exploratory research on the application, opportunities and challenges related to ChatGPT in healthcare. The primary reasons for choosing this research method are as follows: (1) Thematic qualitative text analysis is primarily used for theme-related studies, making it highly suitable for our investigation into the three themes of opportunities and challenges in this study; (2) Thematic qualitative text analysis has developed a relatively systematic theory and methodology, and its effectiveness and rigor have been thoroughly demonstrated in previous research [68,69].

In accordance with the research themes and questions of this study, and by referencing the basic process of qualitative text analysis, the specific steps for conducting thematic qualitative text analysis in this paper were designed as the following five phases.

#### 3.1.1 Develop main topical categories

This article first combed through the relevant articles (including papers, reviews, comments, and opinions) in Google Scholar and PubMed related to ChatGPT applied to healthcare, clarifying the definition of GenAI LLMs and healthcare studied in this article. Then we summarized the synonyms of “applications” (including “using”), “opportunities” (including “benefits” and “potential”), and “challenges” (including “limitations”, “risks”, “problems” and “dilemma”). The use of multiple synonyms for the same keyword in the search was not only for cross-validation of the search results but also for more comprehensive retrieval of valuable information. Articles lacking terms such as “ChatGPT healthcare applications,” “ChatGPT healthcare opportunities,” or “ChatGPT healthcare challenges” and their synonyms in their titles or abstracts were excluded. Additionally, a single article might be utilized in two or even three distinct topics among the aforementioned three.

#### 3.1.2 First coding process

First coding process determined and encoded the search keywords using the main categories (see Table 2). This step was conveniently designed in a sequential manner, meaning that we worked through the text section-by-section and line-by-line from beginning to end, to assign text passages to categories. The assignment of these categories was based on the collective evaluation of the text and the assistance of TermRaider tool, part of the General Architecture for Text Engineering. Then consensus was reached between both authors.

**Table 2:** The summarization of search keywords.

Original word	Search keywords (Code)
Applications	ChatGPT healthcare applications (encoding with “a”); healthcare using ChatGPT (encoding with “b”).
Opportunities	ChatGPT healthcare opportunities (encoding with “c”); ChatGPT healthcare benefits (encoding with “d”); ChatGPT healthcare potential (encoding with “e”).
Challenges	ChatGPT healthcare challenges (encoding with “f”); ChatGPT healthcare limitations (encoding with “g”); ChatGPT healthcare risks (encoding with “h”); ChatGPT healthcare problems (encoding with “i”); ChatGPT healthcare dilemma (encoding with “j”).

#### 3.1.3 Online search

The research used Google browser to search for news articles using the keywords identified earlier, selected a time frame from “November 30, 2022, to April 30, 2023”, selected “news” as the search content type, and used the default “sort by relevance” option from Google. This study chose the Google browser because it is the most commonly used search engine worldwide and has the most comprehensive, authoritative, and up-to-date news reporting content. Based on the following criteria, we searched using the keywords related to the three major topics of application, opportunities, and challenges identified in step two, ultimately obtaining 43, 21, and 25 results that met the criteria respectively. The filtering criteria were as follows: (1) relevance: the news title reflects the theme of ChatGPT in healthcare; (2) effectiveness: the news comes from professionals such as experts and doctors or professional institutions; (3) completeness: the news has a complete structure and includes descriptive cases with the “5w” elements. These three criteria were proposed based on the socially-oriented media theory [70], which suggests that digital media needs to tackle various types of uncertainty. Given the criteria of relevance, effectiveness, and completeness, a news report has a reasonable reference value. Then we utilized the “5w” elements to analyze information in digital media. These elements include: (1) what (i.e., contents/topics of report), (2) why (i.e., motivation of report), (3) how (i.e., types of news), (4) when (i.e., periods of report), and (5) where (i.e., locations of report). The “5w” elements serve as a checklist borrowed from journalism, ensuring that news reports encompass all essential aspects of a story. These elements stem from Lasswell's “5w” Model of communication [71].

#### 3.1.4 Second coding process

Then we coded all of the data using the elaborate category system, being pragmatic and taking the sample size into consideration when determining how many dimensions or sub-categories are suitable for our research (see **Appendix Ⅰ, Ⅱ, &Ⅲ**). The AntConc tool was utilized to help us gather information regarding the mentioned “5W” and compile lists of applications, opportunities, and challenges. These findings are detailed in **Appendix Ⅰ, Ⅱ, &Ⅲ**.

#### 3.1.5 Category-based analysis and results presentation

This phase conducted Category-based analysis of the main categories (presented as *3.2 Data Profile* in this paper), Data display, diagrammatic representations and visualizations (presented as *4. Results* in this paper), and In-depth interpretation of selected cases (presented as *5. Discussion* in this paper).

Category-based analysis of the main categories. This analysis included the “5w” of the news articles, which includes content theme, reporting motivation, reporting form, time span, country or region of origin/originating institution. Analysis was conducted based on every three categories (i.e., applications, opportunities and challenges) with the assistance of AntConc tool, to illustrate the validity and rigor of selected news articles.

Data display, diagrammatic representations and visualizations. Diagrams can be used to gain overviews of sub-categories, and can also be used to compare selected individuals or groups with each other. As a first step, the lead author conducted information extraction, an application form of Natural Language Processing using the software of Python, subsequently obtaining **Appendix Ⅰ, Ⅱ, &Ⅲ**. Then we summarized all of the information in above mentioned Appendix line by line and reached a consensus of the list of applications, opportunities and challenges in Table 6, Table 7, &8. And we performed statistical analysis using Excel to summarize the frequency of relevant items. We concluded with detailed tables and following qualitative analysis.

In-depth interpretation of selected news. Discussion was conducted including the induction of key information and analysis of other insightful viewpoints from the news articles.

### 3.2 Data Profile

The Google browser was used to search for news articles from 1 to 2 May 2023. Information screening was conducted according to the relevance, effectiveness, and completeness criteria mentioned above.

#### 3.2.1 ChatGPT healthcare applications

After conducting standard filtering and removing duplicate reports, 43 news articles were obtained (see **Appendix Ⅰ**). The information statistics of these articles are summarized as follows (see Table 3). Overall, these news articles have good distribution characteristics and high analysis value.

**Table 3:** The profile of search results about ChatGPT healthcare applications.

Statistic	Details
Motivation of Report (Why)	Report the applications: 11 pieces; Explore the applications: 15 pieces; Discuss the effects of applications: 17 pieces (with 15 pieces positive and prudent, 2 pieces negative).
Types of News (How)	News article: 15 pieces; News analysis: 21 pieces; Feature article: 7 pieces.
Periods of Report (When)	Jan.: 3 pieces; Feb.: 10 pieces; Mar.: 18 pieces; Apr.: 12 pieces.
Locations of Report (Where)	U.S.A.: 37 pieces; U.K.: 4 pieces; Australia: 4 pieces; Canada: 1 piece; Chicago: 1 piece; Qatar: 1 piece; India: 1 piece.

Access time: May. 1st, 2023.

In terms of reporting motivation, the focus is mainly on reporting, exploring, and discussing. Among them, the impact of digital media on applications is the most concerned, which is partly because it is most closely related to people's lives, and on the other hand, it reflects the public's general concern about the impact of ChatGPT on healthcare. At the same time, there are relatively few news articles reporting on applications (about one quarter), which reflects that the application of ChatGPT to healthcare is currently in the initial and exploration stage.

The classification of news reports is based on relevant criteria (Full URL: https://libguides.csusm.edu/news/different_news_types, access time: May. 1, 2023). From the statistical results, the news articles used for text analysis in this study have all types of distribution, thus having good statistical significance and analysis value.

The reporting time is all distributed in 2023. The initial report was in January 2023, a little over a month after ChatGPT was released, indicating that generative AI applications represented by ChatGPT quickly attracted people's attention in healthcare. Then the number of related reports has been increasing month by month, reflecting the popularity of ChatGPT applied to healthcare.

The locations of news reports are mainly concentrated in U.S.A. and some European countries. The location of news reports refers to the country where the main subscribers or influencers of the news are located. It mainly focuses on European and American countries because U.S.A. is where ChatGPT was released, and European and American countries have world-leading AI technology levels, strong AI application capabilities, and vast AI application clients. It is worth mentioning that China, which has a competitive AI development and a vast AI application market, has few relevant reports, mainly because ChatGPT has been inaccessible in China since its release.

#### 3.2.2 ChatGPT healthcare opportunities

After conducting standard filtering and removing duplicate reports, 21 news articles were obtained (see **Appendix Ⅱ**). The information is summarized as follows (see Table 4). Overall, the distribution characteristics of these news articles are good and suitable for text analysis.

**Table 4:** The profile of search results about ChatGPT healthcare opportunities.

Statistic	Details
Motivation of Report (Why)	Report the opportunities: 7 pieces Discuss/Explore the opportunities: 14 pieces
Types of News (How)	News article: 10 pieces News analysis: 10 pieces Feature article: 1 piece
Periods of Report (When)	Jan.: 1 piece Feb.: 5 pieces Mar.: 5 pieces Apr.: 10 pieces
Locations of Report (Where)	U.S.A.: 14 pieces U.K.: 3 pieces India: 2 pieces Canada: 1 piece Australia: 1 piece

Access time: May. 1st, 2023.

The four statistical sub-items (listed in the “Statistic” column of Tables 4 and i.e.: Why, How, When, and Where) omitted to be described in detail one by one. It is worth mentioning two points. First, the types of news articles are mainly distributed in the former two (i.e., news articles and news analysis), which reflects that the opportunities brought by applying ChatGPT to healthcare are in a situation where practical promotion and discussion exploration are equally important. Second, the reporting time of news articles is clearly increasing month by month, reflecting the increase in practical cases and the expansion in public attention.

#### 3.2.3 ChatGPT healthcare challenges

After conducting standard filtering and removing duplicate reports, 25 news articles were obtained (see **Appendix Ⅲ**). The information is summarized as follows (see Table 5). Overall, these news articles have covered the dimensions of the last four “w” (i.e., Why, How, When, Where) in the descriptive study of “5w”. Comprehensive analysis shows that all news articles selected in the applications, opportunities, and challenges can well cover all dimensions of the “5w”, which further illustrates the value of mining data information and conducting horizontal and vertical research in this study.

**Table 5:** The profile of search results about ChatGPT healthcare challenges.

Statistic	Details
Motivation of Report (Why)	Report the challenges: 6 pieces Discuss/Explore the challenges: 19 pieces
Types of News (How)	News article:6 pieces News analysis: 15 pieces Feature article: 4 pieces
Periods of Report (When)	Jan.: 2 pieces Feb.: 8 pieces Mar.: 9 pieces Apr.: 6 pieces
Locations of Report (Where)	U.S.A.: 16 pieces U.K.: 3 pieces India: 3 pieces Canada: 1 piece Switzerland: 1 piece Australia: 1 piece

Access time: May. 2nd, 2023.

**Table 6:** Category-based analysis results about ChatGPT healthcare applications.

Stakeholders	List of Applications		Details	Quantity	Sources
Sh.1	Provide medical advice		None.	14	a2, a5, a6, a9, a11, a13, a15, a18, a22, a25, a28; b2, b6, b9.
	Provide diagnosis and triage recommendations		i.e., ask patients questions about their symptoms and medical history to determine the urgency and severity of their condition.	8	a3-5, a7, a9, a18; b10, b11.
	Provide mental health support		None.	8	a1, a4, a9, a10, a12, a22, a25; b11.
	Create symptom checkers		i.e., identity and interpret potential health issues, provide guidance on next steps, and even provide information on self-care measures that a patient can take before seeking medical attention.	7	a1, a4, a10, a14, a15, a17, a27.
	Assist with medication management		e.g., help manage patients' reminders, dosage instructions and potential side effects, provide patients with information about drug interactions, contraindications, and other important considerations that can affect medication management.	6	a1, a4, a17, a22, a29; b4.
	Improve doctor-patient conversations		None.	6	a13, a26, a28; b2, b10, b12.
	Provide remote patient monitoring		None.	5	a4, a8, a17, a25; b11.
	Provide drug information		e.g., provide information about the proper dosage, administration, and storage of medications, as well as potential alternatives for patients who are allergic or intolerant to specific prescriptions.	3	a4; b2, b5.
Sh.2	Streamline repetitive time-consuming administrative processes		e.g., clinician scheduling, patients referrals and credentialing.	18	a3, a5-7, a9, a11, a19, a20, a22, a24, a25, a29; b3, b4, b6-8, b14.
	Support clinal decision		i.e., provide real-time, evidence-based recommendations, suggest treatment options for a specific condition, provide relevant clinical guidelines.	15	a1, a3, a4, a6, a8, a9, a11, a15, a18, a19, a26, a27; b6, b9, b12.
	Finish medical recordkeeping		e.g., generate automated summaries of patient interactions and medical histories, extract relevant information from patients records.	13	a1, a3, a4, a6, a7, a16, a21, a26, a29; b1, b3, b8, b14.
	Provide drug information		e.g., stay informed about new medications, drug recalls, and other important updates in the pharmaceutical industry.	4	a1, a4, a27; b1.
	Process data and extract valuable insights		e.g., develop natural language processing (NLP) algorithms that extract information from unstructured data, and convert it into structured data.	4	a3, a17, a18, a26.
	Assist with medical imaging		None.	4	a16, a26; b12, b13.
	Finish medical translation		i.e., accurately and quickly translate medical jargon, technical terms and common expressions.	3	a1, a4; b4.
	Prevent medical errors		e.g., observe doctors and nurses, compare their actions to evidence-based guidelines and warn clinicians when they're about to commit an error.	3	a8, a14; b12.
Sh.3	Help conduct medical research		None.	7	a5, a10, a15, a26, a29; b1, b11.
	Assist with medical writing and documentation		None.	6	a1, a4, a5, a15, a19; b9.
	Assist with disease surveillance		i.e., monitor global health data, which can give researchers real-time insights into potential outbreaks and facilitate early response efforts.	2	a1, a4.
Sh.4	Develop telemedicine			3	a1, a4; b4.
	Assist with clinical trial recruitment		i.e., identity potential participants who meet the trial's eligibility criteria.	3	a1, a4, a20.
	Help healthcare organizations reach the audiences		None.	1	a3.
	Enable institutions to improve		i.e., enable institutions to place the patient in a wellness-focused environment and help determine ways to keep that person healthy.	1	a18.
Sh.5	Assist students with medical education		e.g., provide instant access to relevant medical information and resources, supporting their ongoing learning and development.	8	a1, a4, a11, a13; b5, b7, b9, b14.
	Revolutionize medical education and practice		None.	1	a23.
	Expand healthcare access in underserved communities		None.	1	b4.

**Note:** The abbreviations used in Table 6 above have the following meanings: **Sh.1:** patients and other healthcare demanders; **Sh.2:** doctors, nurses and other healthcare providers; **Sh.3:** medical experts and other healthcare researchers; **Sh.4:** hospitals, medical groups, policymakers and other healthcare regulators; **Sh.5:** other stakeholders. Sh.5 includes pharmaceutical and medical device companies, digital health startups, funders, and other social groups.

**Table 7:** Category-based analysis results about ChatGPT healthcare opportunities.

Stakeholders	List of Opportunities	Details	Quantity	Sources
Sh.1	Make healthcare more accessible	e.g., for patients who live in rural areas or have difficulty accessing healthcare, for patients with complex or rare conditions that require specialized care.	4	c10; d1, d4; e4.
	Reduce healthcare costs	e.g., searching for symptoms accurately can prompt patients to see a doctor earlier than they typically would and identify a diagnosis and treatment earlier.	3	c1, c2; d3.
	Improve patients care	None.	3	c10; d7; e4.
	Reduce the risk of adverse reactions or other complications	None.	2	c3; e4.
	Bring patients better experience	None.	2	c1; e2.
	Increase the efficiency of patient flow	None.	2	c2, c7.
	Help with patients' adherence to medications	None.	1	c1.
Sh.2	Save time and energy	i.e., reduce the workload for hospital staff.	7	c2, c5, c7; d3, d6; e2, e4.
	Increase the effectiveness and accuracy	e.g., in preventive care delivery, symptom identification and post-recovery care.	5	c2, c5; d2, d3, d7.
	Reduce the risk of errors	None.	1	e4.
	Make healthcare job more promising	None.	1	c4.
Sh.3	Assist with scientific research	e.g., better define underlying mechanisms and therapeutics approaches for diseases, help researchers with the eloquence and fluidity of ChatGPT's response.	3	c9; d1, d5.
	Improve research innovation	None.	2	c5, d2.
Sh.4	Accelerate healthcare system innovation	None.	4	c9; d2; e1, e3.
	Improve the efficiency and effectiveness of healthcare system	None.	3	c9; d3; e4.
	Promote the equity of healthcare system	None.	2	c9, d4.
Sh.5	Initiate medical educational reforms	None.	2	c6, c8.
	Benefit medical device companies	None.	1	e2.
	Encourage collaboration	i.e., encourage collaboration between different sectors and disciplines.	1	d5.
	Positively impact human creativity and productivity	None.	1	e1.
	Used for social purposes	e.g., increase patient engagement in social practice.	1	e2.

**Note:** The abbreviations used in Table 7 above have the following meanings: **Sh.1:** patients and other healthcare demanders; **Sh.2:** doctors, nurses and other healthcare providers; **Sh.3:** medical experts and other healthcare researchers; **Sh.4:** hospitals, medical groups, policymakers and other healthcare regulators; **Sh.5:** other stakeholders. Sh.5 includes pharmaceutical and medical device companies, digital health startups, funders, and other social groups.

The four statistical sub-items (listed in the “Statistic” column of Tables 5 and i.e.: Why, How, When, and Where) omitted to be described in detail one by one. From them, the following trends can be found. The challenges and potential risks brought by the application of ChatGPT to healthcare have attracted widespread attention, mainly reflected in the increasing quantity of news and the general areas of concern.

## 4 Results

**Fig. 1:**
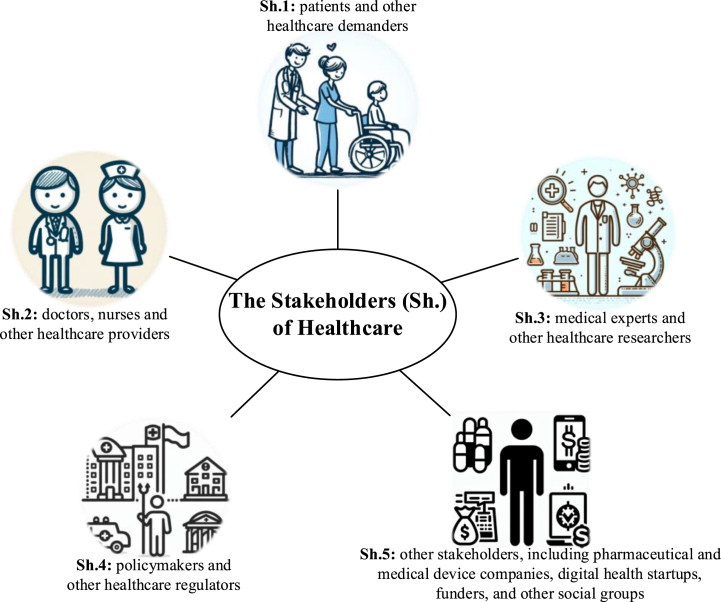
Segmentation of healthcare stakeholders: a fivefold perspective.

**Fig. 2:**
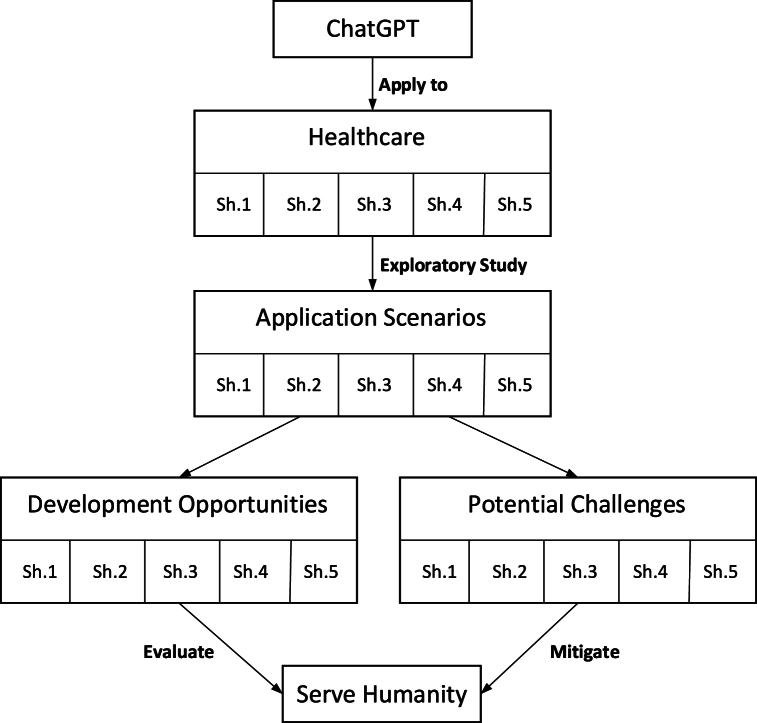
Interconnections among applications, opportunities, and challenges of ChatGPT in healthcare.

This section conducted a category-based analysis of the applications, opportunities, and challenges of ChatGPT in healthcare. The macroscopic and broad concepts need to establish a framework through appropriate classification, thus enabling this research to be organized, comprehensive, and in-depth. Based on the connotation of healthcare and reference to relevant research [[72], [73], [74]], this study summarized the results of the text analysis of these three topics from the perspectives of five different stakeholders, as depicted in Fig. 1. Based on this classification, this study systematically investigated three research topics concerning ChatGPT in healthcare: applications, opportunities, and challenges (see Fig. 2).

### 4.1 ChatGPT healthcare applications

Through text analysis and summarization of 44 news articles related to the application of ChatGPT in healthcare, this research obtained the following results (see Table 6). The research results were classified according to different stakeholders and arranged based on the frequency of news reports. Then explanations/examples of some applications were provided for some listed applications that could be more specific. The table intuitively illustrates the current application of ChatGPT in healthcare, and comprehensively reveals the views and insights of industry experts, clinical physicians, and other professionals regarding ChatGPT used in healthcare. From the results of the table, it can be seen that Sh.1 and Sh.2 are the two main groups involved in the application of ChatGPT in healthcare.

Patients and other healthcare demanders are the direct beneficiaries and risk bearers of ChatGPT's application in healthcare. As a chatbot, direct conversation with patients is the easiest and most practical application. This application has been extended to provide diagnosis and triage recommendations, mental health support, and other uses. Combining ChatGPT with medical devices has created some applications such as symptom checkers and remote patient monitoring. Additionally, ChatGPT, with its machine learning capabilities and LLM, can obtain and integrate cutting-edge information to provide patients with drug information and improve doctor-patient conversations. Overall, ChatGPT has the potential to provide various application services in many scenarios for healthcare demanders.

Doctors, nurses, and other healthcare providers are the direct promoters and indirect bearers of risks of ChatGPT's application in healthcare. Undoubtedly, the application of ChatGPT will relieve healthcare providers from administrative issues and bureaucratic tasks, allowing them more time to complete more professional and valuable work. ChatGPT can also help professionals make clinical decisions with its advantaged characteristics of stability (i.e., occurring few procedural errors), comprehensiveness (i.e., considering comprehensive potential diseases), objectivity (i.e., not being affected by emotions), etc. Additionally, some more accessible and feasible applications are even in practice, such as finishing medical recordkeeping, providing drug information, and finishing medical translation. Furthermore, ChatGPT's technological advancement and rapid updating give itself a significant advantage in applications such as processing data, extracting valuable insights, and assisting with medical imaging compared to analysis AI.

For other healthcare stakeholders, ChatGPT also has significant application value, and many applications have already been realized. For Sh.3, helping conduct medical research and assisting with medical writing and documentation have been widely adopted by researchers. ChatGPT's powerful capabilities of information integration and analysis are bound to drive important research, such as to assist with disease surveillance, to reach a higher research level. For Sh.4, ChatGPT has both advanced applications (e.g., developing telemedicine), and daily applications (e.g., assisting with clinical trial recruitment). For Sh.5, ChatGPT is currently being widely discussed for application in medical education, even for reforming and revolutionizing the system of medical education. Indeed, many applications involving other stakeholders still need to be explored and tested in practice.

Overall, ChatGPT has significant application value and immense prospects for different healthcare stakeholders. However, the risks brought about by inappropriate use, such as equity issues, safety issues, and quality issues (according to **Appendix Ⅰ**, b3), require people to remain vigilant and cautious. Accordingly, experts have proposed that ChatGPT's application in healthcare must comply with the Health Insurance Portability and Accountability Act (HIPAA) in United States (according to **Appendix Ⅰ**, b4), or General Data Protection Regulation (GDPR) in European Union (according to **Appendix Ⅰ**, a25). This, like the application of ChatGPT, is also worth thinking about and researching.

### 4.2 ChatGPT healthcare opportunities

Table 7 presents the results of a textual analysis through summary of 21 relevant news articles on the opportunities that ChatGPT brings to healthcare. Overall, ChatGPT has provided rich opportunities and profound impacts for different stakeholders, involving various aspects such as healthcare delivery ways, interest distribution scheme, healthcare system efficiency, and interdisciplinary cooperation.

For Sh.1, ChatGPT brings about revolutionary changes and opportunities in terms of accessibility, cost, risk, and experience in healthcare. ChatGPT, as a LLM, can also be seen as part of the Internet of Things and a chatbot/digital platform, with the characteristics of network economy and scale economy. Therefore, it has inherent advantages in improving healthcare accessibility and reducing healthcare costs. Furthermore, through various healthcare applications mentioned earlier, ChatGPT can bring multiple benefits to Sh.1, such as cost reduction, experience optimization, and efficiency enhancement.

For Sh.2, the most significant benefit that ChatGPT brings is time-saving/energy-saving and efficiency-improving. By taking on some trivial and tedious workloads, ChatGPT enables Sh.2 to provide valuable healthcare services to places that need them the most, which is equal to rejuvenating medical resources. ChatGPT improves healthcare efficiency and accuracy through clinical applications, which is of great significance to the improvement of the whole healthcare system.

For stakeholders such as Sh.3, Sh.4, and Sh.5, the opportunities that ChatGPT brings are comprehensive and continuously developing, even disruptive. For example, for Sh.4, accelerating innovation is an important opportunity for the breakthrough development of the healthcare system. What's more, for Sh.5, initiate medical educational reforms are the potential opportunity to break the various ills of traditional medical education and match scientific technological progress.

### 4.3 ChatGPT healthcare challenges

This study has obtained the following research results (see Table 8) by summarizing and analyzing 25 news on the challenges that ChatGPT brings to healthcare. These challenges are mainly distributed in ethical challenges such as privacy, employment, legal regulations, academic research, and partly distributed in the technical fields that affect people's lifestyles.

**Table 8:** Category-based analysis results about ChatGPT healthcare challenges.

Stakeholders	List of Challenges	Details	Quantity	Sources
Sh.1	Generate inaccurate/misleading/wrong answers	i.e., This “hallucination” of fact and fictions is especially dangerous regarding things like medical advice or getting the facts right on key historical events. ChatGPT arrives at an answer by making a series of guesses.	14	f1-3, f6, f7, f11, f12, f14-17; g1-3.
	Raise privacy concerns	e.g., train ChatGPT with personal information, accidently share confidential information as a patient.	11	f1, f3, f4, f6, f11, f14, f15, f17; g3; h1, h2.
	Lack of transparency	e.g., don't know the details about how ChatGPT is trained, what data was used, where the data comes from, or what the system's architecture looks like details, making it difficult to know whether it was done lawfully.	5	f3, f9, f13, f18; i1.
	Bring bias/discrimination risks	e.g., ChatGPT has been shown to produce some terrible answers that discriminate against gender, race and minority groups.	4	f2, f3, f11; i1.
	Trigger cybersecurity problems	None.	4	f6, f11; g3; h1.
	Lead to inappropriate procedures	e.g., increase the anxiety of patients, take longer during the appointment because it would be asked for irrelevant information and not have enough time to spend on the relevant information.	3	f7, f12; h1.
	Make patients-providers relationship worse	None.	1	f10.
	Lack of age verification mechanism **(fixed)**	None.	1	f13.
Sh.2	Take jobs from practitioners	None.	2	f3, f11.
	Make patients-providers relationship worse	None.	1	f10.
Sh.3	Raise issues of intellectual property rights	None.	4	f4, f5, f11; h2.
	Prompt concerns about academic and plagiarism	None.	2	f8; j1.
Sh.4	Bring regulatory troubles	None.	2	f13, f15.
	Lack the definition of the right of data subjects	None.	1	f13.
	OpenAI holds all the power	i.e., with great power comes great responsibility. As a private company, OpenAI selects the data used to train ChatGPT and chooses how fast it rolls out new developments. As a result, there are plenty of experts out there warning of the dangers posed by AI, but little sign of things slowing down.	1	f3.
Sh.5	Bring liability issues	None.	2	f11, f12.
	Privacy complicates efforts to build a data pool	None.	1	f18.

**Note:** The abbreviations used in Table 8 above have the following meanings: **Sh.1:** patients and other healthcare demanders; **Sh.2:** doctors, nurses and other healthcare providers; **Sh.3:** medical experts and other healthcare researchers; **Sh.4:** hospitals, medical groups, policymakers and other healthcare regulators; **Sh.5:** other stakeholders. Sh.5 includes pharmaceutical and medical device companies, digital health startups, funders, and other social groups.

Sh.1 is the direct and main stakeholder responsible for the risks brought by ChatGPT in healthcare applications. According to the analysis results, the highest concerned risks are generating inaccurate/misleading/wrong answers and raising privacy concerns. The former involves special groups--patients: the inaccurate or even false answers generated by ChatGPT may be adopted by patients who lack professional medical knowledge, likely to pose a severe threat to patients' life safety and mental health, and even lead to collective social consequences (e.g., misleading public dietary habits, causing public safety incidents, even triggering social unrest). The latter involves highly sensitive data—patients data: any loophole in the collection, transmission, storage, and application of patients data may pose a severe threat to patients safety. The analysis results show that Sh.1 also faces other various forms of challenges, such as the tense doctor-patient relationship caused by patients’ distrust of ChatGPT. However, OpenAI is constantly exploring and solving these practical challenges, such as adding “Data Controls” and “Chat History & Training” functions to GPT-4 to give users more control over their personal information and address privacy threats; and the lack of age verification mechanism has been resolved in this March.

The risks brought by ChatGPT in healthcare are also related to other stakeholders. Among them, Sh.2, as an indirect stakeholder responsible for the risks brought by this new technology in clinical applications, plays a critical role in assessing, judging, and controlling the corresponding risks. Applying ChatGPT to healthcare may bring liability issues: how to determine liability for relevant stakeholders is a daunting challenge.

## 5 Discussion

This article conducts a qualitative text analysis of news articles reporting on GenAI LLMs (i.e., ChatGPT) in healthcare since its release. The method of using multiple synonyms for the same keyword makes this study more systematic, while the data sourced from social media adds to the reliability of this research. Subsequently, the research results provide a comprehensive understanding of the application scenarios, development opportunities, and potential challenges of GenAI LLMs in healthcare. Additionally, these news articles offer insightful viewpoints and in-depth analyses on specific cases and future development trends of this technology in healthcare. To fully explore and utilize the further information in these news articles, this section conducts a discussion from three perspectives: applications, opportunities, and challenges.

### 5.1 Consensus, debates, and facts on GenAI LLMs applications in healthcare

The application of GenAI LLMs in healthcare has sparked extensive discussions. Especially, industry experts and healthcare practitioners have been discussing the impact of GenAI LLMs on the healthcare industry thoroughly. Most of them have a positive and cautious attitude, while a small minority have a negative attitude (see Table 2, with a share of 2/17). After all, in a life-and-death industry like healthcare, the promotion and application of a new technology must rely on its safety, stability, effectiveness, transparency [75], etc. to alleviate people's concerns. Therefore, the acceptance of transformative technologies in the healthcare still has a long way to go [76].

There is currently the following consensus on the application of GenAI LLMs in healthcare. (1) GenAI LLMs application is a practical and useful way to alleviate supply and demand contradictions in healthcare. Healthcare has a serious imbalance between medical resource/service supply and demand: practitioners face cumbersome and heavy daily work, while patients’ demand for high-quality medical services is increasing [51]. The emergence of GenAI LLMs provides an innovative solution to solve this conflict. (2) The application of this transformative technology in the healthcare industry should start with management work, and then to clinical diagnosis work [[4], [77]]. Compared with diagnosis work, management work is more compatible with the design intent of GenAI LLMs as an intelligent chatbot, and its probability of consequences caused by improper application is relatively low (according to **Appendix Ⅰ**, a4 & a18). (3) The role of information technologies (hereafter, ITs) in healthcare is a tool and assistant, not a replacement [78]. The application of GenAI LLMs in healthcare requires human oversight to accomplish some auxiliary work, which cannot fundamentally replace healthcare providers [47].

There are currently debates on the application of GenAI LLMs in healthcare. (1) Whether GenAI LLMs can be applied faster. Some experts suppose that the application of such transformative technology should be accelerated, while others believe that the development and application of such disruptive technology should be suspended, especially in key areas such as healthcare (according to **Appendix Ⅰ**, a29 & b12). Supporters believe that based on Moore's Law, AI technology like GenAI LLMs will double its performance in about two years [79], which means that GenAI LLMs is likely to achieve 30 times the current development in the next decade, so GenAI LLMs should be popularized and applied at a fast pace. Opponents believe that the development of new technologies, especially new technologies with significant social impacts like GenAI LLMs, must be within relevant regulatory frameworks in order to prevent uncontrolled threats to human survival and development [75]. This also means that the development of this technology must be developed after the establishment of perfect policy, laws, and public awareness. (2) Whether GenAI LLMs can be applied to medical education. Some scholars believe that this IT should be prohibited from being applied to medical education, while others believe that this technology should be vigorously promoted for medical education to achieve a revolution in medical education. After ChatGPT successfully passed the U.S. Medical License Examinations in this January, the discussion on ChatGPT's application to medical education reached a climax [65]. Supporters believe that this is a great opportunity to reform the ills of traditional medical education, while opponents believe that this will have an adverse impact on students' learning habits, thinking methods, and other aspects.

However, the following phenomena or facts cannot be denied. (1) GenAI LLMs has been applied to healthcare before the release of ChatGPT and has achieved corresponding effects or purposes, such as medical image recognition and cancer treatment (according to **Appendix Ⅰ**, a26 & b4). (2) The technology was not originally designed for application in healthcare, but it has been practically applied to healthcare. When conversing with GenAI LLMs, it gave an answer that it wasn't designed for healthcare at the first place. The recent release of Google's Med-PaLM, a similar AI model tailored for medicine, and OpenAI's application programming interface, which leverages ChatGPT to build healthcare software, further emphasize the technological revolution transforming healthcare [80]. (3) While the accuracy and efficiency of GenAI LLMs in healthcare have been partially verified in trials, its acceptance among patients is still relatively low [47,76]. (4) The application of this transformative technology in healthcare has received support from many investors, startups, and even tech giants such as 10.13039/100004318Microsoft. Examples include Microsoft's Nuance and DocsGPT (developed based on ChatGPT) (see **Appendix Ⅰ**, b8) [53]. (5) Data is the core issue for this technology used in healthcare [81]. On the one hand, high relevance and up-to-date data are necessary for training sufficiently accurate and effective GenAI LLMs applications. On the other hand, data protection is crucial for the safety of GenAI LLMs application in healthcare. (6) The application of the transformative technology in healthcare is a complicated problem that cannot be considered from only one aspect, such as technology or policy solely.

Many other issues also need to be considered and studied regarding the application of GenAI LLMs in healthcare. For example, whether this IT will cause unemployment issue in healthcare, and if so, how to quantify and research this issue.

### 5.2 Essence, premise, and foundation of GenAI LLMs opportunities in healthcare

The essence of the opportunities that GenAI LLMs brings to healthcare is innovation. This transformative technology represents a shift in the mode of production, as GenAI LLMs begins to engage in certain creative work, changing the way stakeholders in healthcare think about their productive activities [25,51]. With ChatGPT, Sh.1 innovates the way of accessing healthcare; Sh.2 innovates the way of delivering healthcare; Sh.3 innovates the way of researching healthcare; Sh.4 innovates the way of organizing healthcare; Sh.5 innovates the way of studying, funding, implementing healthcare. As a result, these transitions offer broad prospects for healthcare.

The premise for the opportunities GenAI LLMs brings to healthcare lies in its integration with various scenarios in healthcare [19]. These scenarios include off the count monitoring, diagnosis assisting, post-care, late recovery, and etc. (according to **Appendix Ⅱ**, c2, c8, d2 & h4). The IT must be integrated with these scenarios to ensure the professionalism and accuracy of healthcare service [82], empowering healthcare while acting as an auxiliary tool, thus ensures the reliability and safety of healthcare by placing GenAI LLMs within a framework of rules, constraints, and human supervision. Based on specific scenarios, the technology's application in healthcare can be sustained, continuously bringing new opportunities for healthcare development [47].

The foundation for the opportunities that GenAI LLMs brings to healthcare lies in its powerful technological capabilities [26]. Generative AI existed before ChatGPT, but what makes ChatGPT stand out is its sophisticated facilities, powerful computing power, leading algorithms, and other technical advances [18]. These advances are the key for ChatGPT to change production ways and promote productivity development. To deeply impact healthcare, ChatGPT needs to constantly update and match its technological capabilities with application requirements [18]. ChatGPT in essence is a product of technological innovation, and only by continuously refining technological capabilities through practice can it maintain advanced levels and vitality.

### 5.3 Current status and essence of GenAI LLMs challenges in healthcare

People's understanding of GenAI LLMs application is still limited as it is just the initial stage nowadays, and public awareness of its challenges in healthcare is just the tip of the iceberg [15,76]. This is also the main reason why opponents claim to suspend this disruptive technology's training and prioritize regulation. However, it should be recognized that the emergence of a new technology applied to a life-critical industry like healthcare is bound to face many difficulties and bottlenecks, similar to the widespread use of mercury thermometers more than two hundred years ago. An open-minded and cautious attitude, as well as the idea of dynamically discovering and solving challenges through practice, are what people should hold when facing such new technology [15].

The essence of the challenges GenAI LLMs brings to healthcare is uncertainty [35,53]. ChatGPT is developed and owned by private companies (i.e., Open AI), which makes it almost impossible to be completely transparent in algorithm results, generation processes, etc. (according to **Appendix Ⅲ**, f3 & f15). These issues bring various uncertainties. Sh.1 is uncertain whether ChatGPT will bring potential harm to themselves, which leads to a skeptical and negative attitude towards ChatGPT; Sh.2 is uncertain about the operation process of ChatGPT, which raises doubts about its reliability and compliance; other stakeholders are uncertain and even have no basis to judge whether ChatGPT is safe or reliable. The public attitude of suspicion that arises due to these uncertainties depends on deeper understanding and technological advancements to solve.

The challenges of GenAI LLMs in healthcare are closely related to its applications, opportunities, and are of significant importance for studying the development of this technology in healthcare. Of course, there are many perspectives or entry points for conducting such research, this section only provides a framework for discussion.

## 6 Conclusion and the way forward

This article provides a systematic analysis and forward-looking discussion on the applications, opportunities, and challenges of ChatGPT in healthcare from the perspective of digital media. The innovative perspective for filling the gap of systematic research of GenAI LLMs in healthcare fits the research needs. In summary, the release of a powerful tool such as ChatGPT will instill awe, but in healthcare, it needs to elicit appropriate action to evaluate its capabilities, mitigate its harms, and facilitate its optimal use.

This study makes both theoretical contributions and practical implications. Theoretically, this paper provides a direction and theoretical basis for future research topics through a systematic investigation of the application of GenAI LLMs in healthcare. Practically, the study suggests that policymakers should promptly enact relevant laws and regulations to regulate and guide the healthy development of GenAI LLMs in the healthcare industry. Simultaneously, it encourages healthcare practitioners to apply GenAI LLMs with a cautious approach.

Inevitably, this study has the following limitations. Firstly, the data sources were primarily confined to the Google browser and restricted to online news platforms. Although this encompassed a substantial portion of reliable data about ChatGPT, it may not be entirely comprehensive and systematic for information extraction. Secondly, the access time of research data might have a certain interval from the publication date of this article, potentially resulting in a relative lag in our work concerning data.

The application of GenAI LLMs is a large-scale social governance experiment, and its application in healthcare involves innovation and reform in many areas such as medical service delivery methods, medical resource allocation mechanisms, and medical institutional systems [15]. The accumulated data and cases are far from enough to judge whether there will ultimately form a sophisticated healthcare mode. However, it is more likely that humans will develop corresponding ethical rules and regulations as related risks continue to emerge. Future research can be considered from the following two aspects: (1) research content can be explored from a specific healthcare field (e.g., mental health), a specific application scenario, a specific institutional mechanism (e.g., GenAI LLMs' economic benefits allocation mechanism applied to healthcare), or an analysis of GenAI LLMs' influence on healthcare from a technical level; (2) research methods can be conducted through case analysis, empirical analysis, or conceptual analysis. From the general rules of technology applications and the current status of GenAI LLMs’ application, GenAI LLMs is bound to have a profound impact on healthcare. And the specific areas of impact, the speed of impact, how the impact is quantified, etc., need to be studied in practice. Finally, this technology will be developed in a benign way for the benefit of human society.

## Ethical approval

Not applicable.

## Informed consent statement

Not applicable.

## Data availability statement

The original contributions presented in the study are included in the Supplementary material, further inquiries can be directed to the corresponding author.

## CRediT authorship contribution statement

**Rui Xu:** Conceptualization, Data curation, Investigation, Methodology, Software, Writing – original draft, Writing – review & editing. **Zhong Wang:** Formal analysis, Funding acquisition, Investigation, Supervision, Validation, Writing – review & editing.

## Declaration of competing interest

The authors declare that the research was conducted in the absence of any commercial or financial relationships that could be construed as a potential conflict of interest.
